# Automatic Tumor Grading on Colorectal Cancer Whole-Slide Images: Semi-Quantitative Gland Formation Percentage and New Indicator Exploration

**DOI:** 10.3389/fonc.2022.833978

**Published:** 2022-05-11

**Authors:** Shenlun Chen, Meng Zhang, Jiazhou Wang, Midie Xu, Weigang Hu, Leonard Wee, Andre Dekker, Weiqi Sheng, Zhen Zhang

**Affiliations:** ^1^ Department of Radiation Oncology, Fudan University Shanghai Cancer Center, Department of Oncology, Shanghai Medical College, Fudan University, Shanghai, China; ^2^ MAASTRO (Department of Radiotherapy), GROW School of Oncology and Developmental Biology, Maastricht University and Maastricht University Medical Centre+, Maastricht, Netherlands; ^3^ Department of Pathology, Fudan University Shanghai Cancer Center, Department of Oncology, Shanghai Medical College, Fudan University, Shanghai, China

**Keywords:** tumor grading, whole-slide histopathology image, colorectal cancer, deep learning, gland formation, Pathology and clinical outcomes

## Abstract

Tumor grading is an essential factor for cancer staging and survival prognostication. The widely used the WHO grading system defines the histological grade of CRC adenocarcinoma based on the density of glandular formation on whole-slide images (WSIs). We developed a fully automated approach for stratifying colorectal cancer (CRC) patients’ risk of mortality directly from histology WSI relating to gland formation. A tissue classifier was trained to categorize regions on WSI as glands, stroma, immune cells, background, and other tissues. A gland formation classifier was trained on expert annotations to categorize regions as different degrees of tumor gland formation versus normal tissues. The glandular formation density can thus be estimated using the aforementioned tissue categorization and gland formation information. This estimation was called a semi-quantitative gland formation ratio (SGFR), which was used as a prognostic factor in survival analysis. We evaluated gland formation percentage and validated it by comparing it against the WHO cutoff point. Survival data and gland formation maps were then used to train a spatial pyramid pooling survival network (SPPSN) as a deep survival model. We compared the survival prediction performance of estimated gland formation percentage and the SPPSN deep survival grade and found that the deep survival grade had improved discrimination. A univariable Cox model for survival yielded moderate discrimination with SGFR (c-index 0.62) and deep survival grade (c-index 0.64) in an independent institutional test set. Deep survival grade also showed better discrimination performance in multivariable Cox regression. The deep survival grade significantly increased the c-index of the baseline Cox model in both validation set and external test set, but the inclusion of SGFR can only improve the Cox model less in external test and is unable to improve the Cox model in the validation set.

## 1 Introduction

Though there has been intense research interest in molecular prognostic and predictive factors, visual histology examination continues to be the most dependable predictor of survival in patients with colorectal cancer (CRC). Pathologists define the “reference standard” of tumor grading by evaluating visual characteristics, such as nuclear atypia, mitotic activity, and morphological features. New features have been proposed as histopathological prognostic factors, such as poorly differentiated clusters (PDCs) (1), tumor budding (2), and tumor-infiltrating lymphocytes (TILs) (3). In routine clinical practice, prognostication by pathologist-reported overall tumor grade remains the basis for “standard care” in CRC (4–6).

The objective of this study was to evaluate the potential performance of “deep learning” models for individualized survival time predictions directly from histopathology whole-slide images (WSIs) of CRC.

Over 90% of all CRC are adenocarcinoma arising in epithelial cells of colorectal mucosae. The widely used WHO visual grading system defines histological tumor grade based on the density of gland-forming regions (7) on WSI. Tumors are classified as well differentiated, moderately differentiated, poorly differentiated, or undifferentiated, depending on the percentage of the tumor that is gland forming, at >95%, 50%–95%, 5%–50%, and <5%, respectively; however, visual grading has some obvious limitations. First, visual estimation of gland formation (GF) density might be highly subjective (8), and grade assignment thus implies significant inter-observer variability (9–11). Second, quantitative calculation of GF is not presently performed during manual tumor grading due to the lack of tools that work on WSI. Third, the WHO tumor grade alone does not appear to be a sufficiently strong biomarker for personalized therapy decision making.

Numerous publications to date have demonstrated the potential application of deep learning convolutional neural networks (CNNs) for image-based tissue classification and survival prognostication tasks, including on CRC WSI (12, 13). Clinical parameters, such as TIL density and tumor–stroma ratio, have been quantified automatically and accurately using CNNs (12, 14) but fall short of predicting outcome. Other modeling works do predict outcomes of CRC patients, either from WSI or by first computing surrogate indicators (15, 16) such as the tumor–stroma ratio (12) and the proportion of different tissues types present in the WSI (13). These approaches revealed the deep connection between WSIs and outcomes, and we hypothesize that a direct approach applying deep learning on the entire WSIs may provide new prognostic markers beyond the traditional clinical parameters.

To date, no study has shown a survival prediction model on the WHO’s grading system by either a direct or indirect approach. As mentioned above, the most widely accepted method of tumor grading is GF percentage. Therefore, we aim to predict survival outcomes on GF by both the clinical parameter (GF) and the deep learning-based biomarker. It is our hypothesis that the GF percentage can be estimated by an artificial intelligence (AI) model, and subsequently, an AI model can derive a better prognostic marker from spatial heatmaps of GF compared to the estimated GF percentage.

The organization of our work is as follows. First, we developed separate CNN models i) for gland-forming region classification (we hereafter refer to these as tumor differentiation spatial heatmaps) and ii) for automatic segmentation of tissue type regions on the entire WSI. Next, we trained a deep learning Cox proportional hazards model using the aforementioned spatial heatmaps and tissue type segmentation masks as input. We evaluated the prognostic performance among these alternative stratification options for the primary outcome of overall survival (OS). This work combines two well-known The Cancer Genome Atlas (TCGA) public datasets as training and internal validation, and then we added a new private institutional dataset of Chinese subjects as an independent external test set.

## 2 Materials and Methods

### 2.1 Patients and Datasets

Training and validation datasets consisted of TCGA Colon Adenocarcinoma (TCGA-COAD) and Rectum Adenocarcinoma (TCGA-READ) that have been described in detail elsewhere. Briefly, the former contains data from 454 colon and 7 rectosigmoid junction cancer patients, with 983 WSIs (H&E-stained) and an optical magnification factor of 40 (0.25 µm/pixel) or 20 (0.5 µm/pixel). We chose the highest magnification possible to provide detailed data for AI classifiers and sampled tiles that contain enough information for classifying tissues types and GFs, which were confirmed by our pathologists. The latter comprises 364 H&E-stained WSIs derived from 91 rectal, 71 rectosigmoid junctions, and 6 colon cancer patients also with a magnification factor of 40. For the OS model construction, we excluded only WSIs that had no matching survival records. Thus, 857 WSI (out of 983) and 297 WSI (out of 364) from TCGA-COAD and TCGA-READ, respectively, were used for training. Our local institutional dataset consisted of 108 H&E-stained WSIs with a magnification factor of 40 from 108 eligible patients treated from 2008 to 2009. The WSIs in the local dataset were all formalin-fixed paraffin-embedded (FFPE) slides rather than frozen slides in TCGA dataset for validating survival outcomes better. The inclusion criteria of the institutional dataset were as follows: i) follow-up data were available, ii) epithelial tissues covered an area of at least 20% on WSI, and iii) the WSI was clear enough for human grading and free from large contaminated regions.

**Figure 1:**
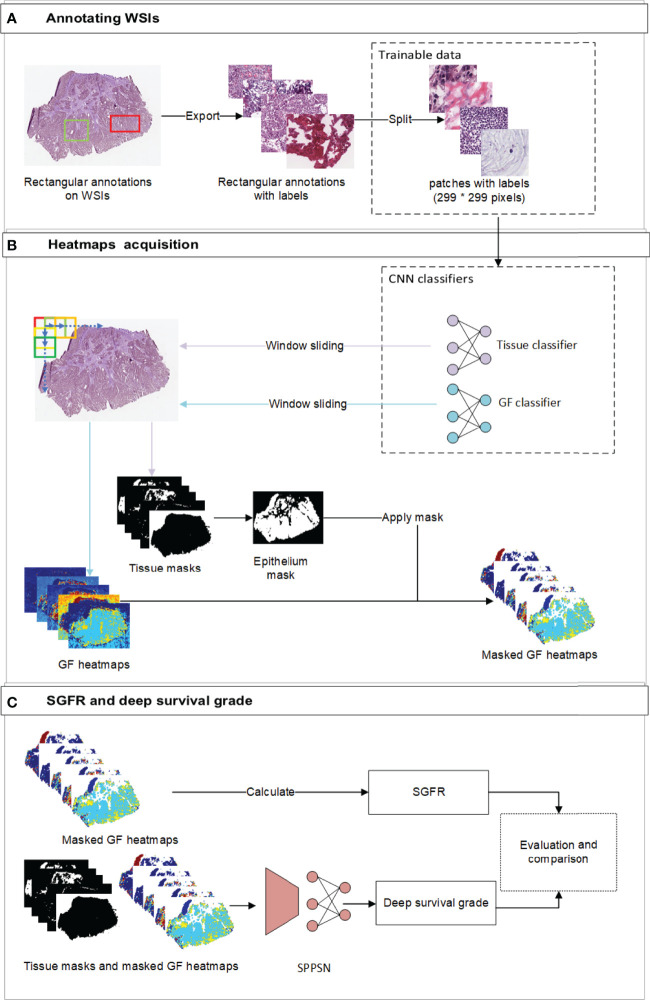
The overview of the workflow. **(A)** The workflow of annotating labels and generating trainable patches. Our pathologists annotated rectangular annotations and related labels from original WSI. We then randomly sampled trainable patches from annotations and balanced the ratio of labels before training. **(B)** the workflow of developing gland formation and tissue category classifiers and generating spatial heatmaps. We used window sliding technique to generate masks of tissue categories and probability maps of tumor gland formation and other tissue types from classifiers. **(C)** The workflow of calculating SGFR and deep survival grade. The epithelium masks were specifically applied to gland formation heatmaps, which were further used in both calculating SGFR and developing SPPSN. WSI, whole-slide image; SGFR, semi-quantitative gland formation ratio; SPPSN, spatial pyramid pooling survival network.

Written informed consent from all institutional patients (050432-4-1911D) and ethics board approval had been obtained. The overall training procedure is as described sequentially in the following subsections and is supported by the illustration in **Figures 1A, B**.

### 2.2 Acquisition of Whole-Slide Image Classification Heatmaps

Spatial heatmaps of tissue classification and GF classification were necessary to obtain the estimated tumor grading and develop the survival prediction models. Two independent supervised deep learning classifiers were trained to generate these spatial heatmaps. To segment new WSI, we implemented a sliding window to span the entire WSI (**Figure 1B**). The reference labels for classification were manually annotated by institutional experts, and internal measures had been taken to reduce inter-observer error. For the tissue classifier, each region of interest (ROI) was classified as epithelium, stroma, immune cells, other tissues, and background. To train the GF classifier, each ROI was then classified by visual inspection as “GF3” (paucity of gland-forming cells), “GF2” (complex or irregular tubules with cribriform morphology glands), “GF1” (simple tubules only), or “X” (normal epithelial cells). The details of developing the above two classifiers, including the reference label annotation method, are reported in **Supplementary Materials S1.1–S1.4**.

### 2.3 Semi-Quantitative Assessment for Gland Formation Ratio

The percentage of the tumor that is gland forming should be calculated to be able to use the WHO stratification system, which can be indirectly assessed by using the abovementioned differentiation maps and tissue category masks. We first focus on the gland regions, which were indicated by category masks, and then estimated the percentage of a gland-forming tumor using the spatial differentiation heatmaps with masks of the gland (**Figure 1C**).

In clinical practice, the estimation of the degree of GF is largely interpretive and lacks standardization for pathologists (8); therefore, the actual GF percentage could not be calculated exactly according to the current WHO definition. So we had to estimate the GF percentage by assigning a semi-quantitative GF ratio (SGFR) in the following manner. We assumed that GF2 regions (complex or irregular tubules with cribriform morphology glands and nuclear polarity partially lost, which sit between gland-forming and non-gland-forming regions) have an arbitrarily assigned weight of 0.5. To spread the weights uniformly, we assigned a weight of 1 to GF1 regions and correspondingly weight 0 to GF3 regions and then excluded all normal tissues. We further assumed the SGFR would be a weighted average of all GF1, GF2, and GF3 in tumor-containing regions as described above will be a reasonably close semi-quantitative approximation of the percentage of gland-forming tumors according to the WHO. The detailed formula of SGFR is provided in **Supplementary Material S2**.

### 2.4 Spatial Pyramid Pooling Survival Network

Spatial pyramid pooling survival network (SPPSN) was trained to predict the hazard of death using information derived from WSI. A “DeepSurv” neural network has been proposed (17) for predicting hazards on time and event data. The input to the network is designed to be a patient’s baseline data. The hidden layers of the network consist of a fully connected layer of nodes, followed by a dropout layer. Therefore, the DeepSurv networks require one-dimensional input data with uniform size. This cannot be applied for our GF spatial heatmaps and category masks, since their shapes obviously change from WSI to WSI. The SPP layer was initially proposed in the field of object detection (18) and has the ability to use arbitrary shape input data. A patient’s data can be transformed into one-dimensional uniform data and thus can be applied to the DeepSurv networks. We thus combined the SPP layer and the DeepSurv networks as an SPPSN and applied it to developing our survival prognostication (**Figure 1C**).

For the input data of the SPPSN, we first applied the epithelium tissue type masks for the GF heatmaps since linear predictor maps due to the GF classifier can only be used on gland-forming regions (**Figure 1C**). The input of the SPPSN consists of i) masked GF maps; ii) masks of stroma, immune cells, other tissues, and background; and iii) thumbnails of the original WSI. These were concatenated as the input layer of the SPPSN.

To develop the OS model, input data derived from both COAD and READ sets combined were randomly split into training and validation in an 80:20 ratio. The SPPSN consists of 1 SPP layer, 3 linear layers, and 1 activation layer. We performed the log-rank test to examine the OS distribution between the validation set and training sets. The p-value was 0.70, which indicated that the OS distribution between the validation set and training set had no statistical difference. The detailed architecture and training parameters of the SPPSN are provided in **Supplementary Material S1.5**.

### 2.5 Statistical Analysis

For the GF classifier and tissue type classifier, we used an area under the curve of the receiver operating curve (AUC) and the confusion matrix to evaluate the model discrimination performance.

For the evaluation of SGFR, we used a Harrell concordance (c-index) and the Kaplan–Meier curve log-rank test on TCGA dataset. To construct the Kaplan–Meier curves, we used three subgroup definitions based on the WHO’s definitions (cutoff points of 0.05, 0.5, and 0.95) and an optimized cutoff (19). Optimized cutoff and WHO were compared for validating SGFR. The optimized cutoff point was obtained by the R package of “maxstat.”

We used a c-index for evaluating the performance of SGFR and deep survival grade from the SPPSN. A Kaplan–Meier curve log-rank test was adopted for performance evaluation as well. A Spearman’s correlation test was applied to determine any correlation between SGFR and deep survival grade. Next, we analyzed common potential OS influencing factors (sex, age, vascular invasion, and American Joint Committee on Cancer (AJCC) stage) in TCGA set and local set in a univariable Cox regression. A multivariable Cox regression of significant factors in the validation set from the last step was developed as the baseline model for comparison. Finally, the SGFR and deep survival grade were separately added into a reference Cox regression model for investigating these grades’ significance and prediction-enhancing power. The performance of the two grades was evaluated and compared using the above multivariable Cox regression models. The calibration curves of Cox regression models were also performed to show the detail.

## 3 Results

The clinical case-mix comparison of included patients with ROI annotations and available follow-up from the three datasets used in this study are given in **Table 1**. Note that the p-value is for the difference between the combined TCGA dataset and the local institutional dataset since the former was used as the discovery set and only the latter was the independent test set.

**Table 1:** Patient characteristics for the datasets of TCGA-COAD, TCGA-READ, and the local institution.

	TCGA-COAD	TCGA-READ	Local dataset	p-Value
***Number of subjects***	451	155	106	-
***Primary cancer***				p < 0.0001
*Colon*	444 (98%)	5 (3%)	104 (98%)	(Fisher’s exact test)
*Rectosigmoid*	7 (2%)	65 (42%)	0 (0%)	
*Rectal*	0 (0%)	83 (54%)	2 (2%)	
*Other*	0 (0%)	2 (1%)	0 (0%)	
***Biological sex***				0.113
*Male*	240 (53%)	87 (56%)	66 (62%)	(Pearson’s test)
*Female*	211 (47%)	68 (3%)	40 (38%)	
***Years of diagnosis***	1998–2013	Not reported	2008–2009	–
***Mean age (range)***	70 (31–90)	67 (31–90)	57 (27–78)	p < 0.0001 (U test)
***Overall staging***				0.136
*I*	71 (16%)	33 (21%)	10 (9%)	(Pearson’s test)
*II*	170 (38%)	49 (32%)	39 (37%)	
*III*	123 (27%)	50 (32%)	37 (35%)	
*IV*	64 (14%)	23 (15%)	20 (19%)	
*Not known*	23 (5%)	0 (0%)	0 (0%)	
*Median survival time (months)*	101	52	Not reached	-
*Median follow-up time (months)*	30	26	87	

TCGA-COAD, The Cancer Genome Atlas Colon Adenocarcinoma; TCGA-READ, The Cancer Genome Atlas Rectum Adenocarcinoma.

### 3.1 Performance of the Tissue Classifier and the Gland Formation Classifier

**Figure 2:**
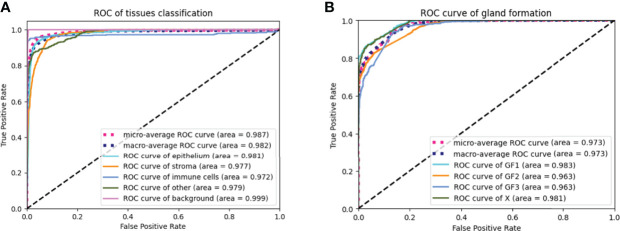
ROC curves of the tissue classifier and the gland formation classifier **(A)** showed ROC curves of the tissue classifier. The AUC were 0.987, 0.982, 0.981, 0.977, 0.972, 0.979 and 0.999 for micro-average ROC, macro-average ROC, epithelium, stroma, immune cells, other and background, respectively. **(B)** showed ROC curves of the gland formation classifier. The AUC were o.973, 0.973, 0.983, 0.963, 0.963 and 0.981 for micro-average ROC, macro-average ROC, GF1, GF2, GF3 and X, respectively.

**Figure 3:**
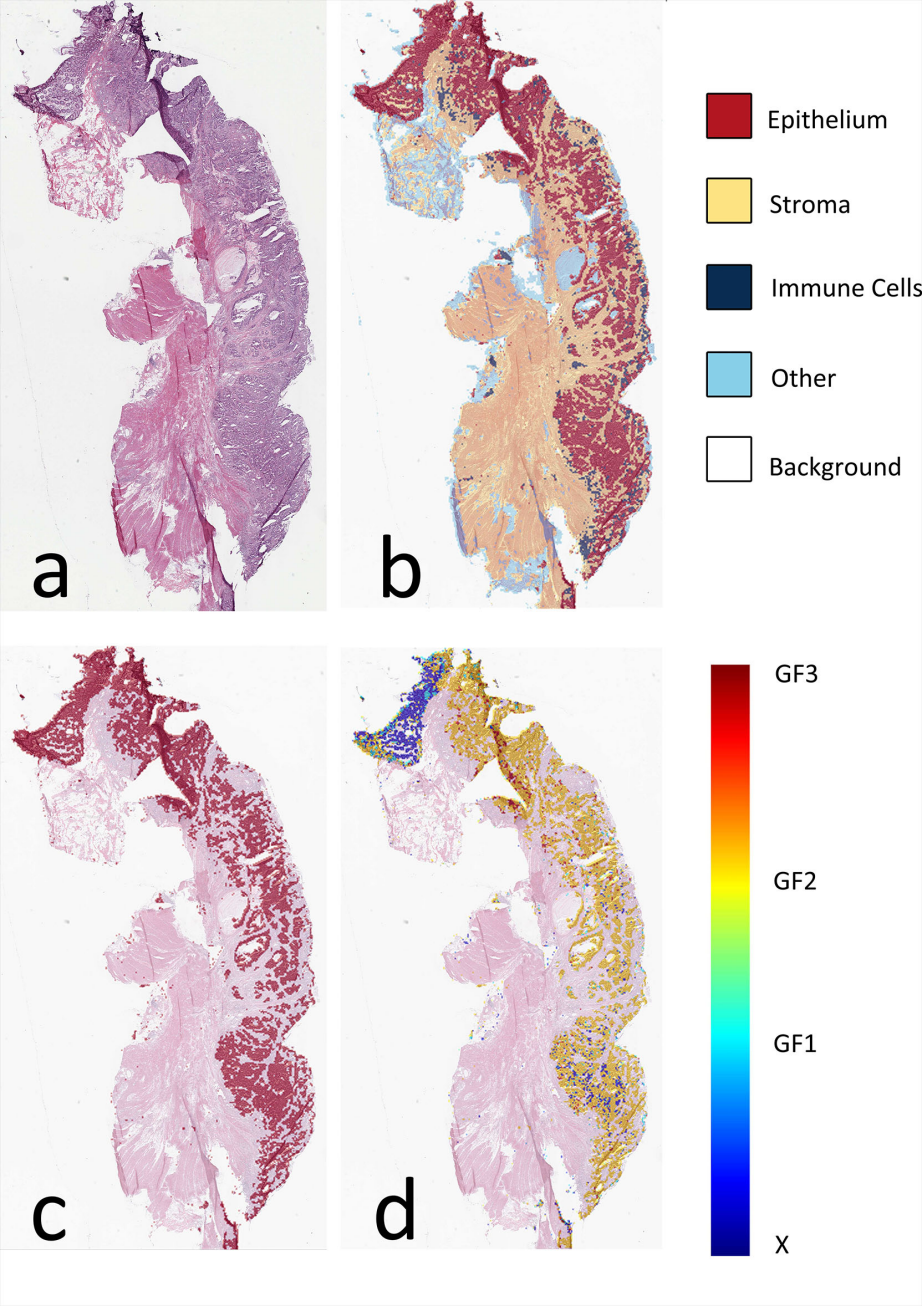
Visual presentation of tissue category and gland formation. **(A)** Thumbnail of original WSI. **(B)** The fusion map of the thumbnail and tissue category map. Different colors represent corresponding tissue categories, and the details can be seen in the right side legend. **(C)** The fusion map of the thumbnail and the epithelium mask selected from panel **(B, D)** The fusion map of the thumbnail and gland formation from the linear predictor on panel **(C)** epithelium mask. WSI, whole-slide image.

The AUC indices of each tissue type of segmentation were calculated, corresponding to discrimination of epithelium, stroma, immune cells, other, and background. The tissue classifier showed strong discriminating ability (AUC = 0.981, 0.977, 0.972, 0.979, and 0.999 for epithelium, stroma, immune cells, other, and background, respectively; micro-average AUC = 0.987, macro-average AUC = 0.982) (**Figure 2A**). Likewise, the AUC indices for gland-forming tumors and normal glandular tissue classification were calculated, corresponding to discrimination between GF1, GF2, GF3, and X. The GF classifier also showed strong discrimination performance (AUC = 0.983, 0.963, 0.963, and 0.981 for G1, G2, G3, and X, respectively; micro-average AUC = 0.973, macro-average AUC = 0.973) (**Figure 2B**). To illustrate the results of tissue classification and gland-forming grades, we show a representative example of a WSI and its maps in **Figure 3**. The original WSI is shown (**Figure 3A**) together with an overlay of the tissue type mask (**Figure 3B**), only the gland-forming regions overlaid on the WSI (**Figure 3C**), and finally, the GF differentiation spatial heatmap within the gland-forming regions overlaid on the WSI (**Figure 3D**). The color on the differentiation heatmap corresponds to the linear predictor of the GF model as shown on the color bar adjacent to **Figure 3D**.

### 3.2 Evaluation and Validation of Semi-Quantitative Gland Formation Ratio on Dataset of The Cancer Genome Atlas

**Figure 4:**
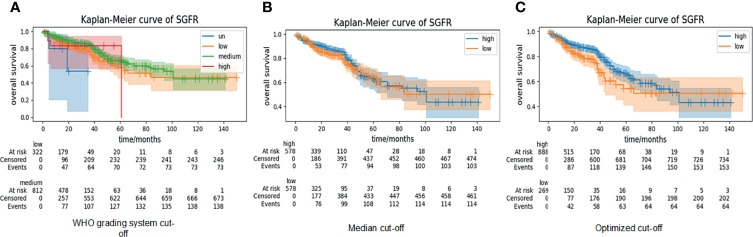
Kaplan–Meier curves of semi-quantitative gland formation ratio (SGFR) with cutoff method of WHO, median, and optimized. According to WHO’s grading system, the WSIs can be grouped into high differentiation (high, SGFR > 0.95), medium differentiation (medium, 0.5 < SGFR < 0.95), low differentiation (low, 0.05 < SGFR < 0.50), and un-differentiation (un, SGFR < 0.05), and the related Kaplan–Meier curves are shown in panel **(A, B)** KAPLAN–Meier curves of WSIs with SGFR higher than median (high) and SGFR lower than median (low). **(C)** Kaplan–Meier curves of WSIs with SGFR higher than optimized cutoff point (high) and SGFR lower than optimized cutoff point (low). WSIs, whole-slide images.

As stated in method section 2.3, we calculated SGFR using the strata of WHO compared with optimized cutoffs. The c-index for SGFR was 0.552, which indicated that the GF percentage possessed poor prediction power. The Kaplan–Meier curves are shown in **Figure 4**. For the WHO’s cutoff method, only a few WSIs belong to the high-differentiation (>0.95) or un-differentiation (<0.05) class. In detail, the number of un-differentiation WSIs and high-differentiation WSIs were 5 and 18, respectively, compared to a total of 1,157 valid WSIs. Therefore, we only focus on medium- and low-differentiation groups whose log-rank test p-values were 0.02. The median cutoff method showed no statistically significant differences (p-value 0.26). The optimized cutoff method indicated only one best point for SGFR (p = 0.01). Optimized cutoff also indicated the optimal threshold of 0.49, which ends up being very close to the WHO’s proposed cutoff point of 0.5, and the WHO’s proposed cutoff point could stratify WSIs well by itself (p = 0.01 *vs.* p = 0.02).

### 3.3 Comparison of Percentage of Gland Formation and Deep Survival Grade

#### 3.3.1 Univariable Analysis

We applied a Cox model and derived Kaplan–Meier curves using the SGFR and deep survival grade on both validation set and test set (**Table 2**). We need to note that the SGFR value is inversely correlated to hazard (i.e., higher GF percentage means higher differentiation status and consequently leads to lower hazard), and thus the hazard ratios of SGFR came out as negative. In the validation set, SGFR showed no significant discriminatory performance, while deep survival grade performed better with a higher c-index and a p-value below 0.05. In the test set, both SGFR and deep survival grade showed significant discriminatory performance, and deep survival grade had a higher c-index of 0.64.

**Table 2:** Univariable Cox models for each metric.

	Validation set			Test set		
Metrics	c-Index	log HR	p-Value	c-Index	log HR	p-Value
Age	0.70	0.065	<0.001	0.56	−0.01	0.38
Sex	0.51	−0.07	0.94	–	–	–
Vascular invasion	0.64	1.12	<0.001	0.57	0.58	0.11
AJCC stage III and IV	0.65	0.91	0.004	0.69	2.43	<0.001
SGFR	0.52	−0.44	0.66	0.62	−7.15	0.07
Deep survival grade	0.64	1.73	0.008	0.64	3.53	0.02

AJCC, American Joint Committee on Cancer; SGFR, semi-quantitative gland formation ratio.

**Figure 5:**
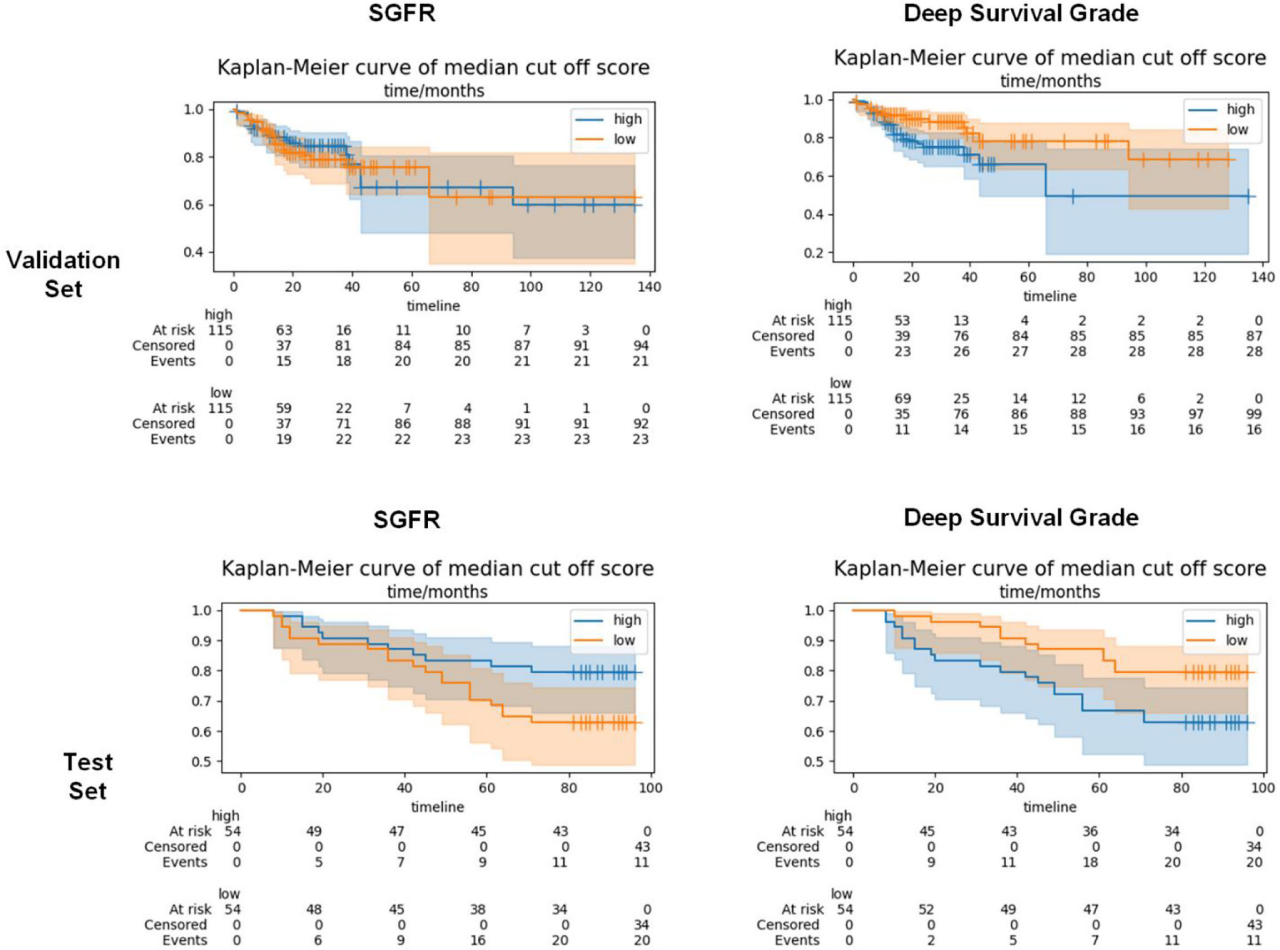
Kaplan–Meier curves of semi-quantitative gland formation ratio (SGFR) and deep survival grade in validation set and test set. SGFR and deep survival grade were grouped into high group (higher than median) and low group (lower than median) and applied Kaplan–Meier curves in both validation set and test set.

For Kaplan–Meier curves, we chose a median cutoff method because the WHO’s cutoff point did not work in deep survival grade and an optimized cutoff point was not suitable for comparison. In the validation set, the Kaplan–Meier curve log-rank p-value of SGFR was 0.69, while that of deep survival grade was 0.02 (**Figure 5**). In the test set, the p-value of SGFR was slightly higher than 0.05 (0.07) and that of deep survival grade was 0.02 (**Figure 5**). In both the validation set and test set, the deep survival grade showed better prediction ability than the GF percentage according to univariable analysis.

#### 3.3.2 Baseline Multivariable Cox Regression Model

We applied a Cox regression model on single factors of age, sex, vascular invasion, and AJCC pathology stage in the validation set (**Table 2**). Then we selected significant factors of age, vascular invasion, and AJCC pathology stage III and IV to build multivariate Cox regression models and apply them on the validation set and test set as baseline models for further reference (**Figure 5**).

#### 3.3.3 Performance Comparison in Multivariable Cox Regression Model

**Figure 6:**
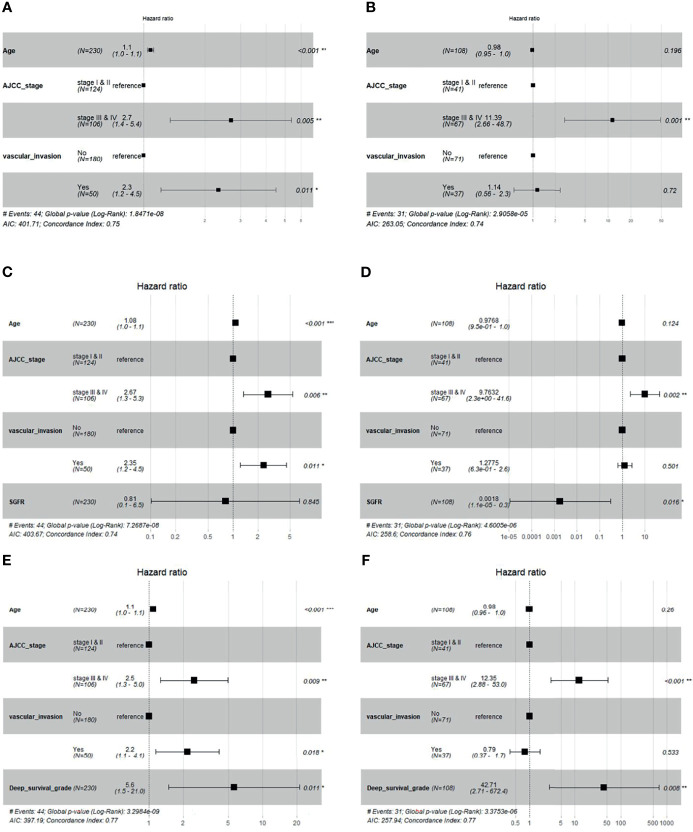
Cox regression forest plot of baseline model, baseline + Semi-quantitative gland formation ratio (SGFR) model and baseline + deep survival grade model. **(A, B)** are the baseline multivariable Cox models of age, AJCC and vascular invasion for validation set and test set, respectively. **(C, D)** are multivariable Cox models of age, AJCC, vascular invasion and SGFR for validation set and test set, respectively. **(E, F)** are multivariable Cox models of age, AJCC, vascular invasion and deep survival grade for validation set and test set, respectively.

We added the GF percentage or deep survival grade separately into the baseline Cox model (Section 2.5) as extra information. The forest plot of Cox regression models is shown in **Figure 6**. When the GF percentage was added to the baseline model (GF enhanced model), it showed no significant performance as an independent hazard factor, and the c-index of the model could not be increased in the validation set, while SGFR enhanced the c-index from 0.74 to 0.76 in the test set. When deep survival grade was added (deep survival grade enhanced model), it showed significant performance as an independent hazard factor, and the c-index was increased from 0.75 to 0.77 in the validation set and from 0.74 to 0.77 in the test set. Hence, deep survival grade possessed an advantage over the GF percentage and enhanced the prediction power of a Cox model based solely on traditional clinical parameters. We also investigated calibration curves of Cox models, and the results are provided in **Supplementary Material S3**.

## 4 Discussion

This study has been a Transparent Reporting of a multivariable prediction model for Individual Prognosis Or Diagnosis (TRIPOD) (20) Type 3 study combining model development and independent validation as one scope of work. We developed and externally validated a CNN model for assigning the GF percentage and a deep survival grade for CRC, based on automated quantitative analysis of WSI. Overall classification accuracy was over 90% for tissue classification and over 85% for GF classification; however, the dominant mode of failure appeared to be the incorrect assignment of GF2 to either normal tissue or high GF tumor slides.

Interpretability of the tissue and GF models was enhanced by providing pathologists with a color-coded composite map as output, as an overlay onto the original WSI. To examine SGFR, we compared the optimized cutoff point of SGFR and the WHO’s proposed cutoff point. Our results indicate that SGFR has moderate prediction ability on stratifying CRC patients whose optimized cutoff point was close to the WHO’s proposed cutoff point of 50%. However, the WHO’s proposed cutoff points of 5% and 95% were unable to be compared in TCGA dataset due to the low sample size. Our optimized cutoff algorithm also indicated that there exists only one cutoff point in TCGA dataset, which supports a two-tiered grading system (21). In external validation datasets, we found that the GF percentage still possessed moderate prediction ability but cannot increase the c-index of the multivariable baseline Cox regression model, which may allude to the limitation of the GF percentage in clinical usage. To discover a new indicator on GF beyond the GF percentage, we developed the SPPSN model, which provided a deep survival grade. Compared to the GF percentage, deep survival grade possessed a higher c-index on itself and could increase the c-index of a baseline clinical Cox model. Therefore, the deep survival grade shows a more promising potential on outcome prediction and even on clinical therapy than SGFR.

The quality of WSIs may illustrate the difference of GF percentage discrimination between TCGA dataset and the local dataset. The overall quality of TCGA’s WSIs was lower than that of a local dataset. The low-quality WSIs such as stained WSIs, WSIs with little regions of epithelium tissue, and highly folded WSIs were common in TCGA set, while WSIs in the local dataset all contained adequate and clear epithelium tissues for tumor grading. Consequently, the GF percentage of the local dataset’s WSIs was more stable and reliable due to better quality.

Our approach has a few advantages over a traditional pathology visual grading workflow. First, a trained machine algorithm is able to exhaustively slide a window of attention over an entire WSI and generate a composite picture of the most probable grade in each window. This helps the pathologist by providing an intuitive visual overview for assigning a tumor grade, along with the heterogeneity within the sample. In effect, the GF model has auto-segmented tumor subregions in the WSI according to their most likely differentiation grade. All of the original WSI information has been retained for closer scrutiny and more precise human review, if so desired.

Calculating the GF percentage was not done within the scope of this present study. This additional feature would be easily performed once the composite maps have been generated. Additionally, this abovementioned workflow will be efficient, objective, and reproducible, which might not always be assured among human observers. There is less chance that a small but significant WSI region might be accidentally overlooked by a human rater. Furthermore, the speed of grading WSIs can be revolutionized by the automatic calculation of SGFR. We also compared the performance of SGFR with human-grading differentiation grade in the test set. The human-grading differentiation grade also shows moderate discriminable ability for stratifying survival outcomes, with a c-index that was close to our SGFR. However, similar to SGFR, the human-grading differentiation grade increases less the c-index of baseline Cox regression model than that of deep survival grade and is also less significant than deep survival grade as an independent factor. Although both SGFR and human-grading differentiation grades were imprecise estimations of the WHO’s differentiation grading system, they still show some value in stratifying survival outcomes. The performance of human-grading differentiation grade can be seen in **Supplementary Material S4**.

Beyond the traditional GF percentage, we also provide a stronger indicator of deep survival grade. Exploring a new indicator from CRC WSIs was previously conducted by several research groups (12, 13, 15, 16). There are two major differences between our work and previous works. First, our SPPSN is the first model that analyzed GF, which can bridge the deep learning output and clinical explanation. Deriving an indicator from a WSI can face the problem of balancing global and local information. The global information is derived from the whole WSI, while the local information uses local regions to represent the whole WSI. Most previous works adopted global information for outcome prediction such as ensemble score of tiles from WSIs (16), weighted classification results (13), or tumor–stroma ratio (12). However, pathologists could assess overall tumor grade either based on a qualitative impression of the sum of global histologic features, or the highest grade of local features observed anywhere in the neoplasm, or the relative proportion of the undifferentiated tumor, or the degree of differentiation along the advancing edge (8). Therefore, both global information and local information were adopted in pathologists’ grading, and simply using one of the two is incomplete. Bychkov et al. included global and local information (15) by long short-term memory method, but the process was still different from pathologists’ grading. Our SPPSN, however, included global information and local information with several levels by SPP layers, and this process was similar to the pathologists’ grading procedure.

A number of limitations remain to be addressed in future work. We examined the errors of misclassification and found that uncertain findings tended to be assigned as GF2. This could be an undesirable side effect of the dataset being highly unbalanced with a much greater proportion of GF2 annotations. We attempted to ameliorate this with majority under-sampling, but it is likely that some vestigial influence persists in the model. Upon closer inspection, these disagreements were also present in pathologists’ manual grade assignments. In real-world clinical practice, the accurate distinction between GF1 and GF2 can also be difficult for human experts. Our present results largely reflect the known difficulty and inherent uncertainty in clinical practice.

Our ground-truth annotations were only provided on so-called “perfect” regions on WSIs, while in real clinical grading, there are common defects such as unintended stains and folded tissues. Our classifiers performed abnormally when there were defects on WSIs, which reduced the classification accuracy of models and consequently influenced GF percentage calculation and deep survival grade prediction.

Of current concern is the misclassification of normal tissue as GF2 tumors. Such false-positive incidents could lead to unnecessary intervention and/or psychological distress to patients. Although the rate of false positives (normal tissue being misclassified as GF1, GF2, or GF3) appears low in this study, it is important to note that such errors can happen. Therefore, cautious clinical commissioning and routine quality assurance of such classification models are needed if they are included in clinical practice (22–24). Although this model has not yet been deployed into routine clinical practice, it has been designed and developed in close collaboration between data scientists and highly experienced clinicians; therefore, it is hoped that a clinical translation gap will be more easily bridged through future work.

In this study, we adopted the WHO’s cutoff point as the reference to validate the estimation of SGFR. However, the cutoff points for the WHO four-tiered schema have not been deeply investigated for their relevance in survival prediction. For example, there exists an alternative two-tiered grading system that has been recommended by a multidisciplinary CRC working group (12), who proposed a 30% cutoff point of gland-forming regions (13). Although our SGFR’s optimized cutoff point seems to agree with that of WHO, the SGFR may be not consistent with real GF percentage on the aspect of distribution.

The grading of CRC WSIs remains to be a complex work due to the existence in the literature of several different grading schemas without widespread acceptance and uniform use of any single system by practicing pathologists (8). However, our research was conducted only on the WHO’s traditional definition of the GF percentage regardless of cytologic features. Tumor budding and TILs, which have been developed as interesting additive prognostic factors in CRC, were not included in our model. Thus, more factors considered in CRC tumor grading can be included in our future works.

There are other clinical factors with potential influence on survival that have not been included in the above model. For instance, different types of surgical and/or drug interventions have not been taken into account. Second, since the WSIs contain the resected tumor, there is little information in the image about the mucosa. Thus, tumor invasion depth and peritumoral morphology will not be available to the CNN to train on as potentially important survival features. No tumor invasion data have been included in the above model.

In conclusion, our study proposed a semi-quantitative estimation of the GF percentage with moderate discriminatory ability on stratifying WSIs’ survival outcomes and an optimized cutoff point similar to the WHO’s cutoff point. This SGFR can be regarded as a good estimation of the GF percentage and has a potential application on automatic CRC tumor grading. We also proposed a novel network architecture (SPPSN) to extract risk information from WSI. Through SPPSN, a new WSI-based predictor (deep survival grade) was created with better performance on stratifying WSIs than that of SGFR. Deep survival grade also showed better performance in a multivariable Cox model compared to SGFR.

## Data Availability Statement

The datasets presented in this article are not readily available because the external dataset of whole-slide images and related patients’ characteristics are not allowed to be shared by the local institution. The model weights, source code, and the annotations on TCGA datasets by pathologists of the local institution are available to share. Requests to access the datasets should be directed to shenlun.chen@maastro.nl.

## Author Contributions

SC designed and performed the experiments, analyzed the data, and wrote the original manuscript. MZ helped collect, annotate, and analyze the data. JW helped design and perform the experiments, analyze the data, and revise the manuscript. MX helped annotate the data and provided pathological knowledge. WH helped in the discussion. LW helped design the experiments, revise the manuscript, and analyze the data and in the discussion. AD helped revise the manuscript and in the discussion. WS helped annotate the data, provide pathological information, and design the experiments. ZZ helped design the experiments, analyze the data, and guide the research presented.

## Conflict of Interest

The authors declare that the research was conducted in the absence of any commercial or financial relationships that could be construed as a potential conflict of interest.

## Publisher’s Note

All claims expressed in this article are solely those of the authors and do not necessarily represent those of their affiliated organizations, or those of the publisher, the editors and the reviewers. Any product that may be evaluated in this article, or claim that may be made by its manufacturer, is not guaranteed or endorsed by the publisher.
