# Supplementary material for: Automatic Tumor Grading on Colorectal Cancer Whole-Slide Images: Semi-Quantitative Gland Formation Percentage and New Indicator Exploration
Source: Front Oncol. 2022 May 11;12:833978. doi: 10.3389/fonc.2022.833978 (PMC9130480; doi:10.3389/fonc.2022.833978)
Supplement: Supplementary file 1 [file DataSheet_1.pdf]

Supplementary Materials

S1 Tissue and gland formation classifiers development and Spatial Pyramid Pooling Survival Networks (SPPSN) development

S1.1 Annotation of gland-forming regions and tissue types

Annotations on gland-forming (GF) regions of interest (ROIs) were provided by our own clinical pathologists with at least 5 years of clinical experience in CRC. Two pathologists working separately and independently first identified ROIs in WSI containing epithelial cells that were at least 800x800 pixels in area (see "WSI" in Fig. 1.A). Each ROI was then classified by visual inspection as one of either "GF3" (paucity of gland-forming cells), "GF2" (complex or irregular tubules with cribriform morphology glands), "GF1" (simple tubules only) or "X" (normal epithelial cells). In any case of inter-rater discrepancy, the ROI was referred to a third pathologist with more than 15 years clinical experience in CRC to adjudicate. As training for a CNN for GF classification, we thus acquired 3436 ROIs with GF region annotated by pathologists from a randomly selected subset of 241 WSI from TCGA-COAD, and 421 ROIs with GF annotated from a randomly selected subset of 28 WSI from TCGA-READ.

Annotations for tissue type (i.e. epithelium, stroma, lymphocytes, slide background or "other") were obtained by manual segmentation by a single radiotherapy technologist. The "other" type referred to tissue that did not belong to one of the other four types, such as muscle, adipose, debris and mucus. This task was deemed reasonably obvious to trained clinical personnel, thus no inter-rater verification was performed for this step. Tissue type segmentations were drawn onto ROIs measuring at least 800x800 pixels in area. For training a tissue type segmentation algorithm, we thus acquired 828 tissue segmentation masks from a randomly picked subset of 74 WSI in TCGA-COAD, and 142 segmentation masks from a randomly picked subset of 10 WSI in TCGA-READ.

S1.2 Image pre-processing

ROIs of 800x800 were still unwieldy for our available computation capacity. We thus randomly sampled "training patches" measuring 299x299 pixels from anywhere within a given ROI. The choice of 299x299 was the default size in the Xception V3 CNN architecture[1]. Training patches were balanced with respect to equal proportions of GF classification by over-sampling for less prevalent GF labels. Data augmentation was used to reduce sensitivity to spatial orientation and image capture conditions; each patch was rotated by a random angle, and then half of these at random were flipped horizontally. Pixel intensities in each patch was also individual perturbed; values were multiplied by a random number between 0.9 to 1.1, and then offset by a random number between -8 and +8. Finally, pixel values were clipped to a maximum of 255. Training and validation were performed exclusively using these resampled and augmented 299x299 patches taken from the combined TCGA-COAD and TCGA-READ. The total number of available patches were randomly split in 90:10 ratio for training and validation.

S1.3 Tissue type segmentation

A tissue-type classifier was trained for 800 epochs, with each epoch containing 2000 batches of batch size 16. An Adam optimizer was used with an initial learning rate of 0.0001 that was progressively reduced by factor 10 in

consecutive 200 epochs. A cross-entropy loss function was used. The CNN architecture was minimally modified from the default Xception V3 network[1]; details of architecture and model training are given in the S1.4. The output layer of the model contained the class probabilities of epithelium, stroma, lymphocytes, slide background and “other” tissue.

To segment new WSI, we implemented a sliding window with size 299x299 and stride length of 149 spanning the whole WSI. In each window position, we recorded the discrete class probabilities. In this manner, five distinct binary masks (one for each tissue type) were generated for each test WSI, with the same dimensions as the WSI. These binary masks were saved for further analysis and subsequent training for a survival prediction model.

We demonstrate architecture of tissue classifier model on Fig. S1. After the architecture of XceptionV3, we added a full connection layer with output size of 5.

S1.4 Gland formation label classification

A GF-classifier was also based on the default Xception V3 CNN and trained with the same hyper-parameters as the aforementioned tissue type model; additional details are provided in the S1.6. However, we derived two types of output from this model; a categorical discrete GF label prediction and a continuous linear predictor (LP; where LP=0.0 implied “X”, LP=3.0 implied “GF3” and so on). The discrete class prediction used to optimize the overall accuracy of label assignment (i.e. X, GF1, GF2 or GF3), while the continuous LP was used to capture the rank relationship between two immediate adjacent grades. The loss function was the sum of two errors, cross-entropy for the discrete category and mean absolute error for the continuous LP.

To test the GF classifier in hitherto unseen WSI, we also used a sliding window technique (see Fig. 1.B) with size 299x299 and stride length of 149 covering the entire WSI. In each window position, we recorded the discrete GF class probabilities as well as the LP value. We then stitched these windows into a composite color map covering the entire WSI, using the LP value as scaling color, then overlaid this onto the WSI to give a direct easy visual reference for pathologists to evaluate. These GF color maps were saved for further analysis and subsequent training for a survival prediction model.

We demonstrate architecture of GF classifier model on Fig. S2. After the architecture of XceptionV3, we added a full connection layer with output size of 4. Then this output was further added with 4 full connection layers with output size of 4 and a full connection layer with output size of 1 for grading and this output was also applied with softmax for classification. We calculate the loss of categorical cross entropy for task of classification and the loss of mean absolute error for task of grading and the sum of two loss are defined as loss function for training.

S1.5 Model architecture and details of Spatial Pyramid Pooling Survival Network (SPPSN)

We demonstrate architecture of SPPSN on Fig. S3. There were 3 phases in SPPSN. The first phase is input preprocessing. In this phase, an original WSI was translated into tissue maps by tissue classifier. The tissue maps included 5 channels of epithelium, stroma, immune cells, other and background. The original WSI was also translated into GF maps by GF classifier which included 5 channels of GF1, GF2, GF3, X probability and linear predictor. The epithelium channel in tissue maps was separately applied on GF maps to generate masked GF maps. The remaining 4

channels of tissue maps (stroma, immune cells, other and background channel) along with thumbnail of original WSI were concatenated into masked GF maps to obtain input data with 12 channels for SPPSN training. The second phase was SPP layer. The input data were segmented into spatial bins which had sizes proportional to the input data size. The proportions were 1, 1/2, 1/4 and 1/6 in this study. In each spatial bin, we pooled the responses of each filter by maxpooling approach. Thus 1-dimension features can be generated by pooling for each bin proportion and final representation features of input data was obtained by adding these 1-dimension features. The last phase was survival network. Three linear layer blocks with shape of 32, 16 and 1 constituted the survival network. Each linear layer block included a drop out layer with drop out ratio of 0.5, a linearlayer, a batch normalization layer and a ReLu activation layer.

The input to the SPPSN was given a 50% chance of flip image transformation, and all were randomly rotated by either 0°, 90°, 180° or 270°. In a single epoch, the training set was repeated 4 times and flipped/rotated as described. The model was trained with learning rate of 0.0001, SPP pooling shape of (6, 4, 2, 1), with L2 penalty of 0.01, minimum batch size of 64, dropout rate of 0.5 and epoch size of 20. The Adam optimizer and ReLU activation was used.

S2 Calculation for semi-quantitative gland formation percentage and comparison

We may generalize the SGFR to alternative weightings in the following manner. The probabilities given by the GF-classifier that a given tile t contains the label g is denoted as PGF (t = g) , where g has one of the allowed labels of "GF1", "GF2" or "GF3". The expression $\sum_g w_g * P_{GF}(t = g)]$ thus represents a weighted sum of gland-forming class probabilities such that the weights are defined by the factor w_g . The probability that the same tile t contains any type of gland-forming tissue is $P_{GF}(t \neq 'X')$, where the aforementioned label 'X' denotes normal epithelial cells. Similarly, the probability that the tissue type in the tile t is actually "epithelium" according to the tissue segmentation will be $P_{TS}(t = 'G')$. We estimate a semi-quantitative gland-forming tumor ratio (SGFR) as a weighted average of gland-forming class probabilities over every available tile t, on condition that the tissue type is "gland" and the type of gland-forming region is not normal epithelial cell 'X'. All this is expressed in Equation (1). Note that there are three slightly different calculation approaches according to AI prediction, we chose the best estimation in survival analysis for evaluation, validation and comparison.

$$\text{SGFR} = \frac{\sum_t [P_{TS}(t = 'G') * P_{GF}(t \neq 'X') * \sum_g w_g * P_{GF}(t = g)]}{\sum_t P_{Ti}(t = 'G') * P_{GF}(t \neq 'X')} \qquad (1)$$

Besides the definition of SGFR based on probability which is also referred to PSGFR, a category SGFR (CSGFR) was also proposed based on category result of tissue classifier and GF classifier. In probability approach, a tile was regarded as mixture of different classification results with responding probability while in category approach, a tile was regarded as one classification outcomes according to maximum of classification results. The formula (2) illustrate the CSGFR.

$$\text{CSGFR} = \frac{\sum_{\{t|argmax\{P_{TS}(t)\}='G' \wedge argmax\{P_{GF}(t)\}\neq'X'\}} w_g}{\sum_{\{t|argmax\{P_{TS}(t)\}='G' \wedge argmax\{P_{GF}(t)\}\neq'X'\}} 1} \quad (2)$$

In GF classifier, a linear predictor was obtained and thus a new SGFR based on deep linear predictor (DSGFR) can be calculated. The linear predictor may reveal more detailed status between GF classifications thus it's worthy to be investigated. The formula (3) illustrate the definition of DSGFR.

$$\text{DSGFR} = \frac{\sum_{\{t|predictor(t)\geq predictor(t='GF1')\}}[P_{TS}(t='G') * w_{gl}]}{\sum_{\{t|predictor(t)\geq predictor(t='GF1')\}}[P_{TS}(t='G')]} \quad (3)$$

In formula (3) predictor(t) denotes the score that tile t predicted by linear predictor, predictor(t='GF1') denotes score of GF1 that predicted by predictor. The linear predictor predicts score from 0 to 3, score 0 means normal tissue,1 means GF1, 2 means GF2 and 3 means GF3, respectively. Therefore, if score higher that 1 (the score of GF1) means the tissue was tumor indicated by predictor. The w_{gl} denotes the linear weight to linear predictor, in mathematic expression w_{gl} = [1 - (predictor score – 1) / 2]. In this expression score 3 responding to weight of 0 which indicate non-gland formation, score 3 responding to weight of 1 which indicate tumor with gland formation.

The performance of all these 3 approaches were conducted in TCGA dataset. The c index for PSGFR, CSGFR and DSGFR were 0.552, 0.548 and 0.548 which indicated SGFR possessed moderate prediction power. The Kaplan-Meier curves were shown in (Fig. S4). For WHO's cut-off method, only few WSIs belongs to high-differentiation (>0.95) or un-differentiation (<0.05). In detail, number of un-differentiation WSIs and high-differentiation WSIs were 5 and 18, 5 and 31, 5 and 32 corresponding to probability differentiation grade, category differentiation grade and deep differentiation grade compared to total valid 1157 WSIs. Therefore, we only focus on Kaplan-Meier curves of medium- and low-differentiation groups (p=0.02, 0.02, and 0.03 for PSGFR, CSGFR and DSGFR, respectively, log-rank test). The median cut-off method showed no statistic significant (p=0.26, 0.27, and 0.28 for PSGFR, CSGFR and DSGFR, respectively, log-rank test). The optimized cut-off method indicated only one cut-off point for all three differentiation grades and the related groups were significant (p=0.01, 0.01, and 0.02 for PSGFR, CSGFR and DSGFR, respectively, log-rank test). Optimized cut-off method also provided the cut-off point of 0.49, 0.51 and 0.50, respectively. The optimized cut-off points were very close to WHO's proposed cut-off point and WHO's proposed cut-off point could stratify WSIs well itself. In general, these three approaches achieved to similar performance in c-index and log-rank P of Kaplan-Meier curves and we choice PSGFR that had relative best performance in c-index to represent SGFR.

S3 Calibration curves of multivariable Cox models

The 60 months calibration curves of baseline model, gland formation enhanced model and deep survival grade enhanced model were shown in Fig.S7. As can be seen in Fig.S7, calibration curves of deep survival enhanced model were closer to perfect calibration curve than other models in both validation set and test set.

S4 Performance of human histological grade in test set

In the local institutional dataset, we were able to examine Kaplan-Meier survival curves split according to a three-part histological grade assigned by human experts - high, medium and low differentiation. These grades were assigned by

pathologists based on human visual inspection only of whole WSIs. The results shown in Fig. S6. The c-index of human grading was 6.2 which was also similar to our semi-quantitative gland formation ratio. The spearman correlation test also indicate the correlation between human grading and SGFR, the correlation coefficient was 0.234 with p value of 0.015. A indicates statistically significant stratification between medium and low differentiation, and was confirmed by log rank tests with p value of 0.02. There existed 4 subjects in group high differentiation but all of them were not observed endpoint events thus no curve of group high differentiation was presented in Fig. S5. In multi-variable Cox analysis with clinical features (Fig. S5), this grading did not indicate a hazard ratio statistically significantly different from 1. The c-index here was 0.76, which was the same discrimination performance as for age, AJCC stage and vascular invasion alone. The human grading shared the same power of enhancing multi-variable Cox model with SGFR.

Reference

1. Chollet, F. *Xception: Deep Learning with Depthwise Separable Convolutions*. 2016. arXiv:1610.02357.

Figure Legends

Fig. S1 Architecture of tissue classifier

Fig. S2 Architecture of gland formation (GF) classifier

Fig. S3 Architecture of Spatial Pyramid Pooling Survival Network (SPPSN). In input data processing stage, channels of gland in tissue heatmaps will mask on gland formation heatmaps. The rest 4 channels of tissue heatmaps together with masked formation heatmaps and thumbnails of original WSIs were put into SPPSN. The shapes of SPP layer were 1, 2, 4 and 6. Finally a deep survival networks were added behind the SPP layer.

Fig. S4 Kaplan-Meier curves for different SGFR algorithms via different cut-off methods Kaplan-Meier curves of probability differentiation grade, category differentiation grade and deep differentiation grade with cut-off method of WHO, median and optimized. The columns represent different differentiation grades, and the rows represent different cut-off method.

Fig. S5 Kaplan-Meier curves of the local institutional dataset for human grading. Kaplan-Meier curves of low differentiation, medium differentiation and high differentiation by human experts in local institutional dataset.

Fig. S6 Hazard ratio of the Cox model combined with human grading in the local institutional dataset. Multivariable cox model of age, AJCC stage, vascular invasion and differentiation provided by human experts.

Fig. S7 The 60 months calibration curves of baseline model, gland formation enhanced model and deep survival grade enhanced model.

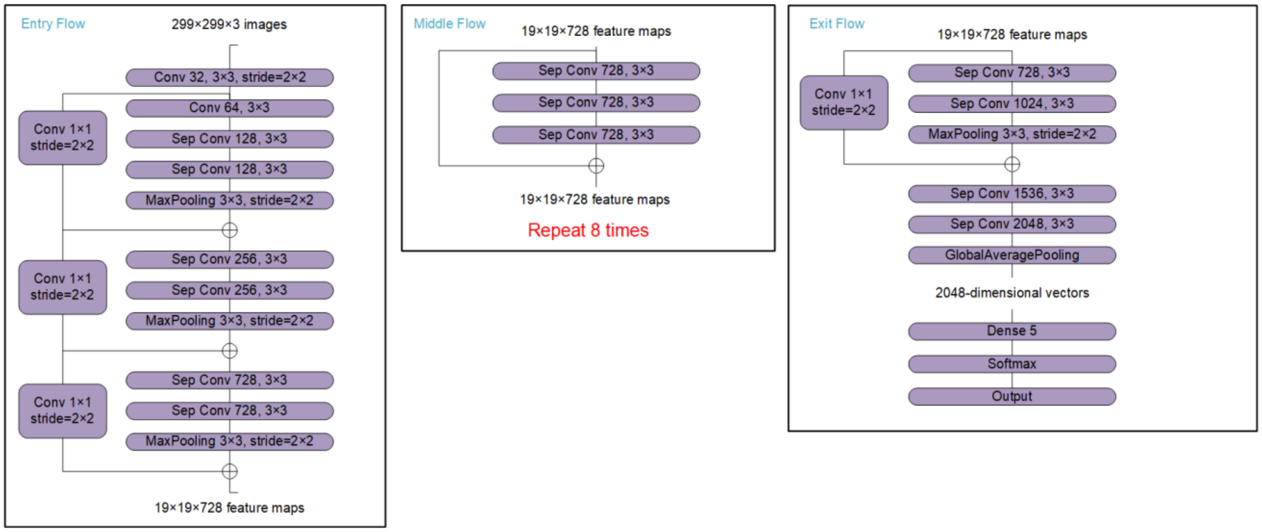

Fig. S1 Architecture of tissue classifier

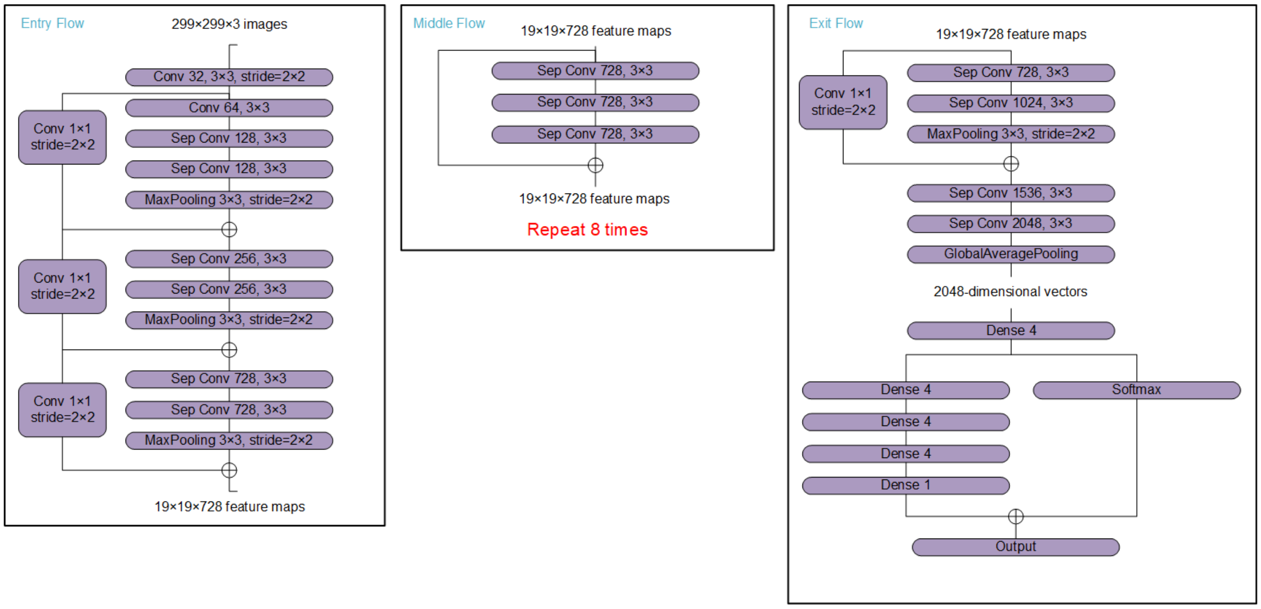

Fig. S2 Architecture of gland formation (GF) classifier

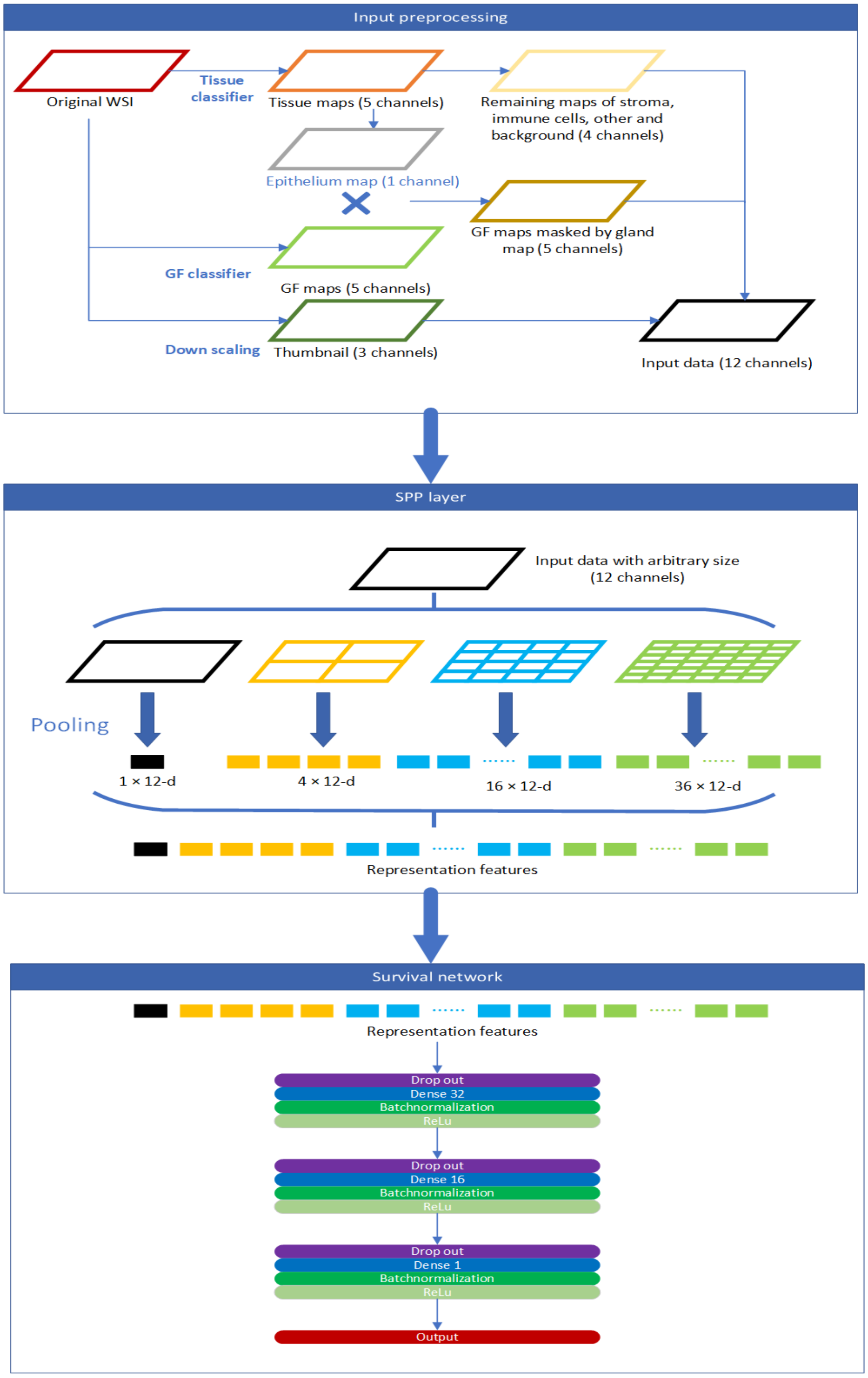

Fig. S3 Architecture of Spatial Pyramid Pooling Survival Network (SPPSN).

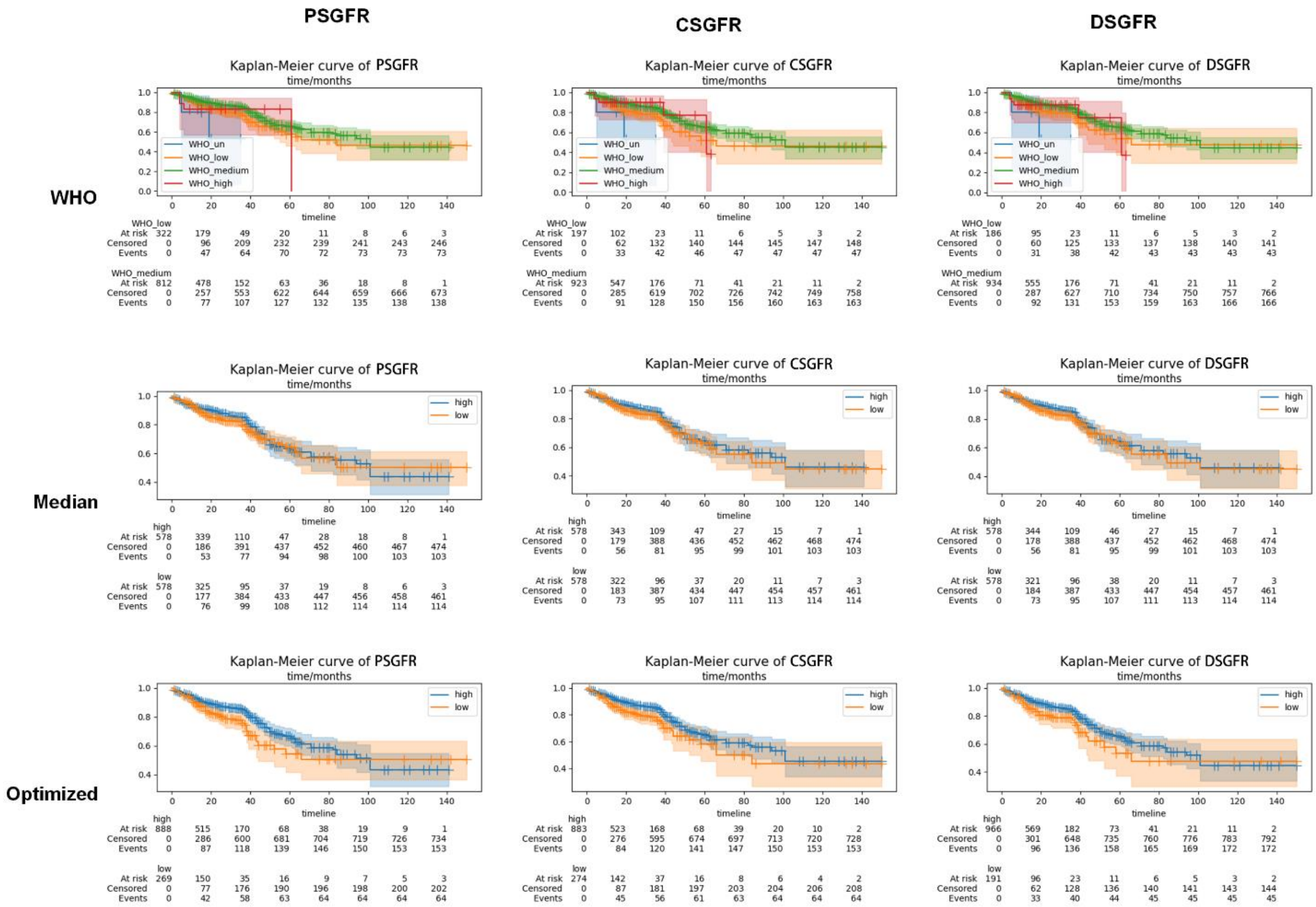

Fig.S4 Kaplan-Meier curves for different SGFR algorithms via different cut-off methods

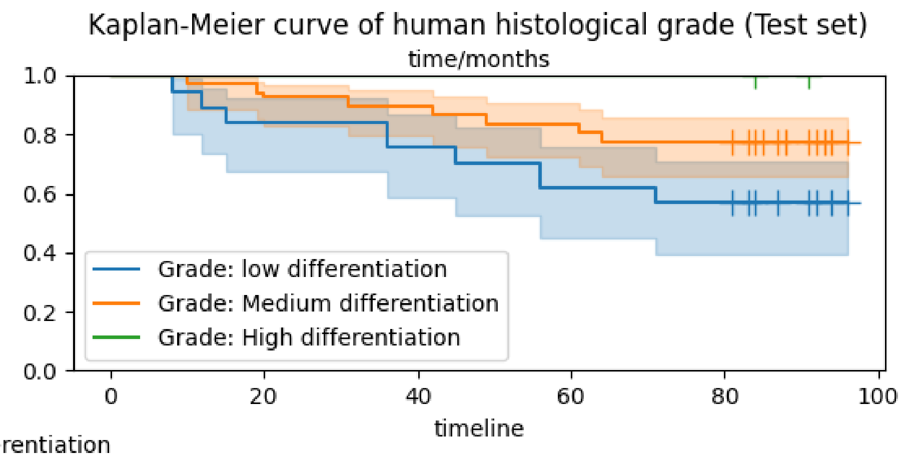

Grade: low differentiation						
At risk	37	31	28	23	21	0
Censored	0	0	0	0	0	21
Events	0	6	9	14	16	16

Grade: Medium differentiation						
At risk	67	62	60	56	52	0
Censored	0	0	0	0	0	52
Events	0	5	7	11	15	15

Fig. S5 Kaplan-Meier curve of the local institutional dataset for human grading.

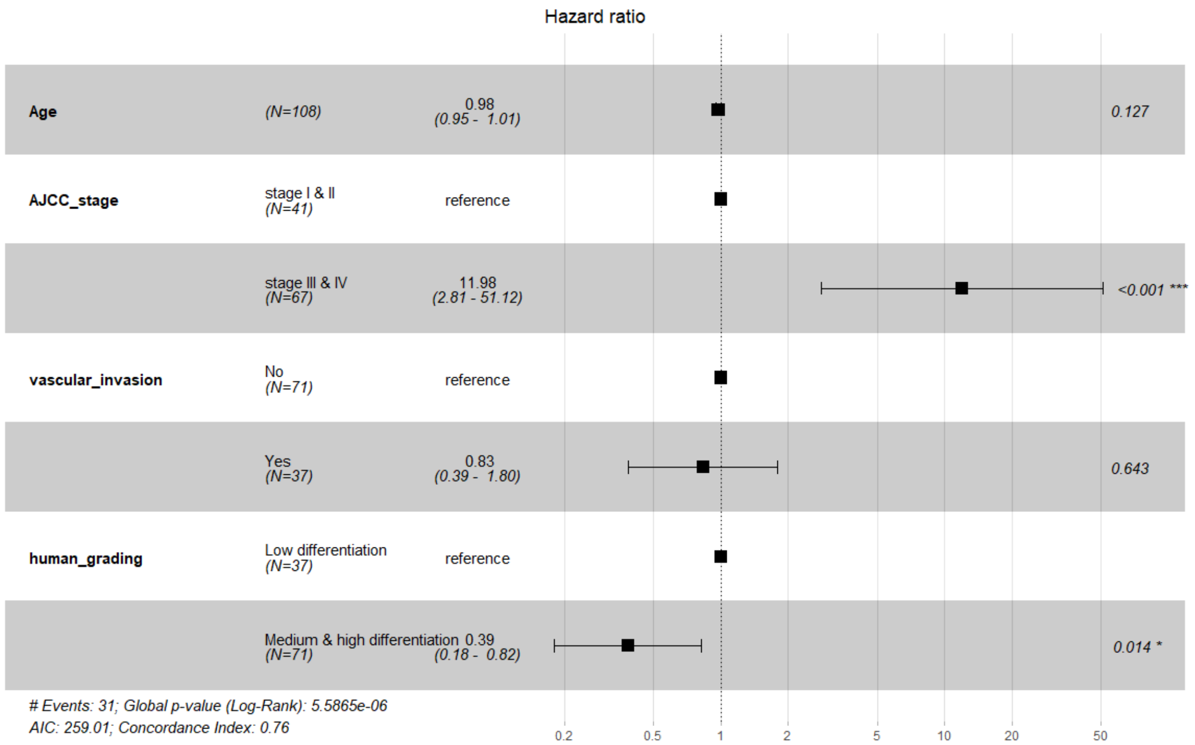

Fig. S6 Hazard ratio of the Cox model combined with human grading in the local institutional dataset.

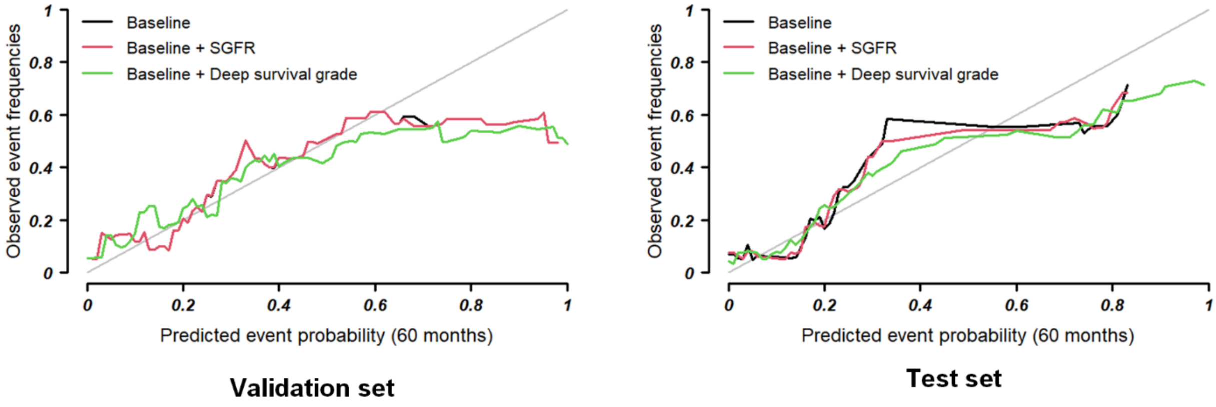

Fig.S7 The 60 months Calibration curves of baseline model, baseline + gland formation model and baseline + deep survival model.
